# Crosstalk between cardiomyocytes and noncardiomyocytes is essential to prevent cardiomyocyte apoptosis induced by proteasome inhibition

**DOI:** 10.1038/s41419-020-03005-8

**Published:** 2020-09-19

**Authors:** Fang Guo, Chen-Chen Zhang, Xi-Hui Yin, Ting Li, Cheng-Hu Fang, Xi-Biao He

**Affiliations:** 1 Shanghai University of Medicine and Health Sciences Affiliated Zhoupu Hospital, 201318 Shanghai, China; 2 Laboratory of Stem Cell Biology and Epigenetics, School of Basic Medical Sciences, Shanghai University of Medicine and Health Sciences, 201318 Shanghai, China; 3 Department of Cardiology, Yanbian University Hospital, 133000 Yanji, China; 4 Department of Pediatrics, Yanbian University Hospital, 133000 Yanji, China; 5 International Peace Maternity and Child Health Hospital, Shanghai Jiao Tong University School of Medicine, 200030 Shanghai, China

**Keywords:** Apoptosis, Cardiovascular diseases

## Abstract

Heart is a multi-cellular organ made up of various cell types interacting with each other. Cardiomyocytes may benefit or suffer from crosstalk with noncardiomyocytes in response to diverse kinds of cardiac stresses. Proteasome dysfunction is a common cardiac stress which causes cardiac proteotoxicity and contributes to cardiac diseases such as heart failure and myocardial infarction. The role of crosstalk between cardiomyocytes and noncardiomyocytes in defense of cardiac proteotoxicity remains unknown. Here, we report a cardiomyocyte-specific survival upon proteasome inhibition in a heterogeneous culture consisting of cardiomyocytes and other three major cardiac cell types. Conversely, cardiomyocyte apoptosis is remarkably induced by proteasome inhibition in a homogeneous culture consisting of a majority of cardiomyocytes, demonstrating an indispensable role of noncardiomyocytes in the prevention of cardiomyocyte apoptosis resulting from proteasome inhibition. We further show that cardiomyocytes express brain natriuretic peptide (BNP) as an extracellular molecule in response to proteasome inhibition. Blockade of BNP receptor on noncardiomyocytes significantly exacerbated the cardiomyocyte apoptosis, indicating a paracrine function of cardiomyocyte-released extracellular BNP in activation of a protective feedback from noncardiomyocytes. Finally, we demonstrate that proteasome inhibition-activated transcriptional up-regulation of BNP in cardiomyocytes was associated with the dissociation of repressor element 1 silencing transcription factor (REST)/ histone deacetylase 1 (HDAC1) repressor complex from BNP gene promoter. Consistently, the induction of BNP could be further augmented by the treatment of HDAC inhibitors. We conclude that the crosstalk between cardiomyocytes and noncardiomyocytes plays a crucial role in the protection of cardiomyocytes from proteotoxicity stress, and identify cardiomyocyte-released BNP as a novel paracrine signaling molecule mediating this crosstalk. These findings provide new insights into the key regulators and cardioprotective mechanism in proteasome dysfunction-related cardiac diseases.

## Introduction

Ubiquitin-proteasome system (UPS) is critical for the protein quality control in the heart. UPS dysfunction is associated with a wide range of cardiac pathologies including cardiac hypertrophy, familial cardiomyopathies, chronic heart failure and myocardial ischemia-reperfusion injury^1–5^. Reduced proteasome activity is also a characteristic of cardiac aging, which further exaggerates the functional decline of the heart and triggers numerous age-related diseases^6,7^. UPS dysfunction induced by proteasome inhibition disrupts intracellular protein turnover and degradation of misfolded, oxidized, and damaged proteins, thereby causing cellular proteotoxicity. Protective cellular response to proteasome inhibition is critical for cell survival, as the continued presence of proteotoxicity usually leads to apoptosis in different cell types including cardiac cells^1–3,8^. Cardiomyocytes are regarded particularly susceptible to proteotoxicity as they are post-mitotic cells, which are unable to dilute the proteotoxicity through asymmetric division^2^. Indeed, a number of studies have provided in vitro evidence of proteasome inhibition-induced apoptosis in cultured cardiomyocytes^9–12^. However, systemic proteasome inhibition in vivo prefers cardiomyopathies rather than cardiomyocyte apoptosis^5,9,13^. In addition, cardiomyocyte apoptosis appears to be far more rare than noncardiomyocyte apoptosis in diseased hearts^14,15^. Given that cardiomyocytes communicate with a variety of noncardiomyocytes in the myocardium in response to pathophysiological stresses^16^, crosstalk between cardiomyocytes and noncardiomyocytes is likely to play a protective role in cardiomyocytes’ response to proteasome inhibition, the evidence of which remains lacking.

Cardiomyocytes utilize various kinds of survival strategies in defense of different cardiac stresses^17^. One of these strategies is through cell-cell communication. Within the heart, for instance, IL-33 is produced by cardiac fibroblasts as paracrine signal to alleviate cardiomyocyte hypertrophy in pressure overload and myocardial infarction^18^. Extracellular vesicles are secreted by cardiac progenitor cells (CPCs) to inhibit cardiomyocyte apoptosis after myocardial infarction^19^. Between organs, natriuretic peptides (NPs) including atrial natriuretic peptide (ANP) and brain natriuretic peptide (BNP) are specifically secreted by stressed cardiomyocytes to exert cardioprotective function^20,21^. This cardioprotective effect is mainly attributed to cardiomyocytes as endocrine cells and NPs as hormones acting on remote organs such as kidneys and adrenal glands to regulate blood pressure and fluid homeostasis^22^. Furthermore, in clinical treatment of cardiovascular diseases such as heart failure and myocardial infarction, NPs are also used as diagnostic and therapeutic tools for their diuretic and natriuretic roles^23,24^. However, whether NPs have any direct cardioprotective function on local environment remains unclear. On the other hand, animal experiments using BNP knockout mice and cell experiments using exogenous BNP have demonstrated paracrine effects of BNP on cardiac fibroblasts to reduce ventricular fibrosis and on CPCs to promote proliferation and differentiation, respectively^25,26^. The comprehensive roles of BNP in the cardiovascular system are far from fully understood.

In fetal and adult heart, the expressions of ANP and BNP are normally maintained at basal levels but significantly increased in response to developmental cues or cardiac stresses^27^. This regulation occurs at the transcriptional level. *Nppa* and *Nppb*, genes encoding ANP and BNP respectively, are paralogous genes positioned in a distance of only several kilo base pairs of DNA sequence to each other in mammalian genomes^28^. In fetal heart development, both genes are highly expressed for chamber myocardium differentiation. After birth, *Nppa* expression is strongly decreased whereas *Nppb* expression remains in adult ventricle. Upon hypertrophy and heart failure, both genes are drastically up-regulated^27^. Their similar expression pattern in development and diseases have led to the hypothesis that the *Nppa*-*Nppb* cluster shares common cis-regulatory elements and transcriptional regulatory mechanisms^29^. However, stress-stimulated expression patterns of *Nppa* and *Nppb* are not always spatio-temporally overlapping. For instance, in animal models of acute myocardial infarction and cardiac hypertrophy, expressions of *Nppb* and *Nppa* were not simultaneously up-regulated^30,31^. Genome-wide studies have also identified distinct stress-responsive regulatory DNA elements at proximal and distal regions of two gene loci^27^. These studies suggest that the molecular mechanisms regulating *Nppa* and *Nppb* expression are highly complex and diverse in response to different stresses.

Here we provide evidence of a proteasome inhibition-activated crosstalk between cardiomyocytes and noncardiomyocytes, which has an essential role in preventing cardiomyocyte apoptosis from proteasome inhibition-induced proteotoxicity. Using multiple cell culture approaches, we demonstrate that this crosstalk is initiated in cardiomyocytes responding to proteasome inhibition, mediated by cardiomyocyte-released extracellular BNP, and feedbacked by noncardiomyocytes, thereby providing the first evidence of BNP as a novel paracrine signaling molecule for cardioprotection. We further identify repressor element-1 silencing transcription factor (REST) repressor complex, which have been shown to regulate both *Nppa* and *Nppb* expressions upon hypertrophic stimuli^32–34^, as key regulators of proteasome inhibition-activated BNP expression in cardiomyocytes, which may serve as pharmaceutical targets for cardioprotective purposes. These findings reveal a new adaptive survival strategy of cardiomyocytes and provide insights into the paracrine communication between cardiomyocytes and noncardiomyocytes in response to proteotoxicity.

## Materials and methods

### Ethics

All animal experiments were approved by the animal ethics committee of Shanghai University of Medicine & Health Sciences and have been performed in accordance with the ethical standards laid down in the 1964 Declaration of Helsinki and its later amendments.

### Cell culture and treatment

Neonatal mouse pups (postnatal day 1) were supplied by Shanghai Jiesijie Experimental Animal Co. Mouse pups were decapitated and the ventricles were quickly dissected under a microscope. For heterogeneous cardiac cell culture, the tissues were washed two times with cold phosphate buffered saline (PBS), enzymatically digested with papain (Sigma) and Accutase (Thermo Scientific) for 10 mins, followed by the second round of enzyme digestion with Dispase I and Collagenase IV (both from Sigma) for another 10 mins, and mechanically pipetted into single cells and plated onto laminin-coated culture coverslips, plates or dishes. Cells were applied to tests after 5 days when they reached full confluency. For homogeneous cardiomyocyte culture, similar cell extraction and dissociation procedure was performed using enzymes from Pierce primary cardiomyocyte isolation kit (Thermo Scientific) according to manufacturer’s manual. A cardiomyocyte growth supplement from the kit was added to reduce noncardiomyocyte growth during cell culture periods. Cells were applied to tests after 5 days when no cell growth was obviously observed. For noncardiomyocyte culture, heterogeneous cell culture was passaged once by Accutase and mechanical pippetting to eliminate all cardiomyocytes. The remaining noncardiomyocytes were re-plated and allowed to grow for another 5 days. All three kinds of cell cultures were grown in Dulbecco’s modified Eagle medium supplemented with 10% fetal bovine serum and 1% Penecillin/Streptomycin. Cells were incubated in 5% CO_2_, 37 °C incubator. To induce proteasome inhibition, 10–50 μM MG132 (Sigma), 1–10 μM Bortezomib (Selleck) or 1 to 10 μM Delanzomib (Selleck) were added for various periods of time. To induce cathepsins and calpain inhibition, 1 to 10 μM Pepstatin A (Santa Cruz), 4–40 μM Leupeptin hemisulfite (Sigma) or 0.5–5 μM E-64-D (Santa Cruz) were added for 24 hrs. To block BNP receptor natriuretic peptide receptor A (NPR-A), 2 μg/mL A71915 (Tocris) was pre-treated one hour before MG132 treatment. To inhibit HDAC activity, a cocktail of 500 nM Trichostatin A and 100 μM valproic acid (both from Sigma) was pre-treated one hour before MG132 treatment. For 5-bromo-2′-deoxyuridine (BrdU) labeling, 10 μM BrdU (Sigma) was added to cells for 30 mins, replaced by two times PBS wash and fresh culture media.

### Fluorescence immunostaining analysis

Cells were fixed with 4% paraformaldehyde for 20 mins, permeabilized and blocked with PBS with 0.3% Triton-X100 and 1% bovine serum albumin for 40 mins, then incubated with primary antibodies diluted with blocking solution at 4 °C overnight. Alexa Fluo series of second antibodies (Thermo Scientific) were applied accordingly for one hour at room temperature. Cells were finally mounted in 4′,6-diamidino-2-phenylindole (DAPI) and examined using fluorescence microscope (Leica DMi8). For BrdU-labeled cells, cells were fixed with cold methanol for 15 mins on ice, rehydrated with PBS for 10 mins, incubated with 2 M hydrochloric acid for 45 mins and neutralized with sodium borate for 15 mins before first antibody incubation. The first antibodies include rabbit anti-active caspase 3 (a-cas3), rabbit anti-BNP, rabbit anti-ANP, mouse anti-sarcomeric α-actinin (SAA; all from Abcam), rabbit anti- discoidin domain-containing receptor 2 (DDR2; Immunoway), mouse anti-smooth muscle actin (SMA; Cell Signaling Technology), Lectin from tomato, FITC conjugate (Sigma), rabbit anti-BrdU (Abcam), and rabbit anti-GATA binding protein 4 (GATA4; Proteintech).

### Western blotting analysis

Cells were lysed for 30 mins in lysis buffer with protease inhibitor cocktails (Sigma). Lysate concentration was measured with Pierce BCA assay kit (Thermo Scientific) and 5 μg of protein was separated by SDS-polyacrylamide gel electrophoresis and transferred to nitrocellulose membrane. The membrane was blocked with 5% skim milk (Cell Signaling Technology) for 1 h at room temperature, then incubated with first antibodies including mouse anti-β-actin (Cell Signaling Technology) and rabbit anti-ubiquitin (Proteintech) overnight at 4 °C. The membranes were washed three times with Tris-buffered saline and Tween-20 followed by incubation with the peroxidase-conjugated anti-mouse or anti-rabbit secondary antibodies (both from Millipore) for 1 h at room temperature.

### Measurement of BNP concentrations

After brief centrifuge and filtration, cell supernatants from 24-well plates were harvested and applied to BNP enzyme immunoassay kit (Sigma) according to manufacturers’ instructions. A standard curve was used for the calculation.

### Real-time PCR analysis

RNA was extracted, reverse-transcribed into cDNA using commercially available kits (TAKARA), then amplified, and applied to real-time PCR analyses (Roche). The comparative cycle threshold method was used for quantification. The forward and reverse primers for *Nppb* are respectively 5′-AGGGAGAACACGGCATCATT-3′ and 5′-GCCAGGAGGTCTTCCTACAA-3′. The forward and reverse primers for *Nppa* are respectively 5′-CTGCTTCGGGGGTAGGATTG-3′’ and 5′-TAGATGAAGGCAGGAAGCCG-3’.

### Chromatin immunoprecipitation (ChIP)

Cells were cross-linked with 1% paraformaldehyde for 15 mins and Chromatins were sheared into an average 200–400 bp in length by sonication (Diagenode) and immunoprecipitated with following antibodies: rabbit anti-REST (Abcam), rabbit anti-CoREST (Upstate), rabbit anti-SIN3A, rabbit anti-HDAC1 (both from Proteintech). Immunoprecipitated DNA fragments were collected by magnetic beads (Active Motif), purified, and subjected to real-time PCR using primers specific to regions spanning predicted REST binding site on *Nppa* and *Nppb*. The forward and reverse primers for NRSE_b are respectively 5′-GCCTGGAAATCGGTTGAGGA-3′ and 5′-TTCCCAAACAACACCCACCA-3′. The forward and reverse primers for NRSE_a are respectively 5′-TCTAGTGGGGTCTTGCCTCT-3′ and 5′-GTCTGTCCTTGGTGCTGAAGT-3′. Data were normalized to values of the input DNA. REST binding site were identified using the Jaspar database (http://jaspar.genereg.net/).

### Cell counting and statistics

Immunoreactive or DAPI-stained cells were counted in at least 10 random regions of each culture coverslip using an eyepiece grid at a magnification of ×50 to ×400. Data are expressed as mean ± S.E.M. of three to five independent cultures. Statistical comparisons were made using Student’s *t*-test or one-way ANOVA with Tukey’s post hoc analysis (Graphpad Prism).

## Results

### Proteasome inhibition does not induce cardiomyocyte apoptosis in heterogeneous cardiac cell culture

**Fig. 1:**
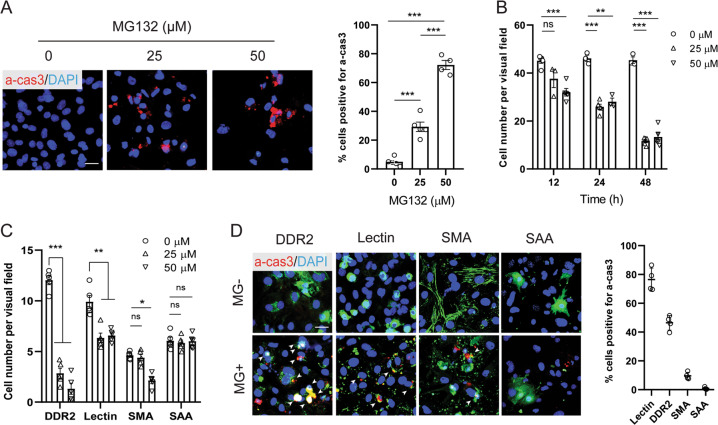
Differential apoptotic responses to MG132-induced proteasome inhibition in a heterogeneous cardiac cell culture containing cardiomyocytes, cardiac fibroblasts, endothelial cells and vascular smooth muscle cells. **a** Representative images and quantification of cells positive for apoptosis marker active caspase 3 (a-cas3; red) after 24-h treatment of various concentrations of MG132. Cell nuclei were labeled with 4′,6-diamidino-2-phenylindole (DAPI; blue). **b** Time- and dose-dependent total cell loss in response to MG132 evidenced by quantification of DAPI + cells. **c** Cells were treated different concentrations of MG132 for 24 h and each cell type population was labeled by immunostaining and quantified. Cardiac fibroblasts were labeled with discoidin domain-containing receptor 2 (DDR2), endothelial cells with Lectin, vascular smooth muscle cells with smooth muscle actin (SMA), and cardiomyocytes with sarcomeric α-actinin (SAA). Note that only the number of SAA + cardiomyocytes was not decreased by MG132. **d** Representative images and quantification of cells labeled with a-cas3 (red) and each cell type marker (green) with or without 24-h treatment of 25 μM MG132. White arrow heads indicate double positive cells. Note that only SAA + cardiomyocytes were not co-labeled with a-cas3 within MG132 treatment. Cell numbers were counted in 10 random areas of each culture coverslip using an eyepiece grid at a magnification ×100. *n* = 4 independent culture. Data represent mean ± S.E.M. *P < 0.05, **P < 0.01, ***P < 0.001, ns not significant; one-way ANOVA with Tukey’s post hoc analysis. Scale bar represents 20 μm.

To investigate the proteotoxic effect of proteasome inhibition on different cardiac cell populations, we employed a heterogeneous cell culture extracted from neonatal mouse hearts which consisted of 10.54 ± 2.06% SAA + cardiomyocytes, 10.13 ± 1.59% SMA + vascular smooth muscle cells, 26.43 ± 2.83% DDR2 + cardiac fibroblasts, and 29.02 ± 2.09% Lectin+ endothelial cells (Supplementary Fig. 1A). Treatment of proteasome inhibitor MG132 caused a drastic increase of poly-ubiquitinated proteins within 24 hrs (Supplementary Fig. 1B), confirming the efficiency of proteasome inhibition. A significant decrease of total cell number was induced by MG132, in correlation with a large number of cells expressing end-stage apoptosis marker a-cas3 (Fig. 1a). In addition, longer time of treatment resulted in more severe cell death (Fig. 1b), demonstrating a reciprocal relationship between total cardiac cell survival and the extent of proteasome inhibition. Immunostaining analysis with each cell type marker showed that DDR2 + cardiac fibroblasts and Lectin+ endothelial cells were significantly lost when exposed to MG132 at 25 μM, whereas the number of SMA + smooth muscle cells was not significantly decreased until the concentration reached 50 μM. Surprisingly, the SAA + cardiomyocytes were totally spared even at 50 μM (Fig. 1c). Co-labeling of a-cas3 with each cell type marker further confirmed that the top two cell populations undergoing apoptosis were Lectin+ endothelial cells (76.43 ± 4.02%) and DDR2 + fibroblasts (46.43 ± 2.53%). In contrast, a minority of SMA + smooth muscle cells and no SAA + cardiomyocytes expressed a-cas3 in response to MG132 (Fig. 1d). Of note, although the decrease of cell number of SMA + cells was not significant at 25 μM, a condensed and disorganized cytoskeleton morphology of SMA + cells was clearly observed, whereas the α-actinin morphology of SAA + cells remained intact at 50 μM (Fig. 1d), suggesting a higher vulnerability of vascular smooth muscle cells to proteasome inhibition than cardiomyocytes. Treatment of two other proteasome inhibitors Bortezomib and Delanzomib resulted in similar outcomes as MG132 (data not shown). Taken together, these data demonstrated an unexpected result that cardiomyocytes were highly resistant to proteasome inhibition-induced proteotoxicity when co-cultured with other cardiac cell populations.

### Proteasome inhibition induces cardiomyocyte apoptosis in homogeneous cardiomyocyte culture

**Fig. 2:**
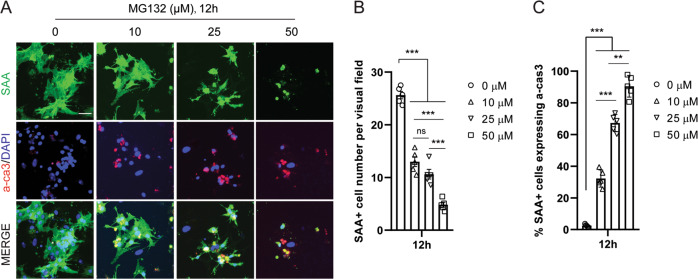
Proteasome inhibition induced severe apoptosis in a homogeneous cell culture of cardiomyocytes. **a** Representative images of sarcomeric α-actinin (SAA) + cardiomyocytes (green) positive for apoptosis marker active caspase 3 (a-cas3) after 12-h treatment of various concentrations of proteasome inhibitor MG132. In this cell culture over 90% of cells without MG132 treatment were SAA + cardiomyocytes. Note that the morphology of cardiomyocytes started to change at MG132 concentration as low as 10 μM and was completely disrupted at 50 μM. **b**, **c** The number of SAA + cells and SAA + cells expressing a-cas3 were quantified, respectively. Cell numbers were counted in 10 random areas of each culture coverslip using an eyepiece grid at a magnification ×100. *n* = 5 independent culture. Scale bar represents 50 μm. Data represent mean ± S.E.M. **P < 0.01, ***P < 0.001, ns not significant; one-way ANOVA with Tukey’s post hoc analysis.

The cardiomyocyte-restricted survival effect in heterogeneous cell culture could have derived from an intrinsic resistance to proteotoxicity or indirectly from an anti-apoptosis support from neighboring noncardiomyocytes. To distinguish this, a homogeneous cardiomyocyte culture was modified from the heterogeneous cell culture to maximize the cardiomyocyte population to about 90%. Cells were treated with the same extent of MG132 as in heterogeneous cell culture. Surprisingly, in contrast to the complete survival of cardiomyocytes through 24-h treatment of 50 μM MG132 in heterogeneous cell culture, more than half of cardiomyocytes were unable to survive a 12-h treatment of 10 μM MG132 in homogenous cell culture (Fig. 2a, b). Expression of a-cas3 was significantly increased in MG132-treated cardiomyocytes, indicating that they were undergoing severe apoptosis (Fig. 2a, c). Moreover, all the cardiomyocytes were either lost or greatly damaged in morphology by 50 μM MG132 within 12 hrs (Fig. 2a), while cardiomyocytes in heterogeneous culture remained intact by same extent of treatment for 24 hrs (Fig. 1c, d). Consistently, treatment of Bortezomib and Delanzomib also caused severe cardiomyocyte apoptosis in homogenous cell culture (data not shown). Collectively these results strongly indicated that cardiomyocytes were intrinsically vulnerable to proteasome inhibition, and the survival effect of cardiomyocytes in heterogeneous cell culture was derived from co-cultured noncardiomyocytes.

### Proteasome inhibition induces BNP expression in cardiomyocytes

**Fig. 3:**
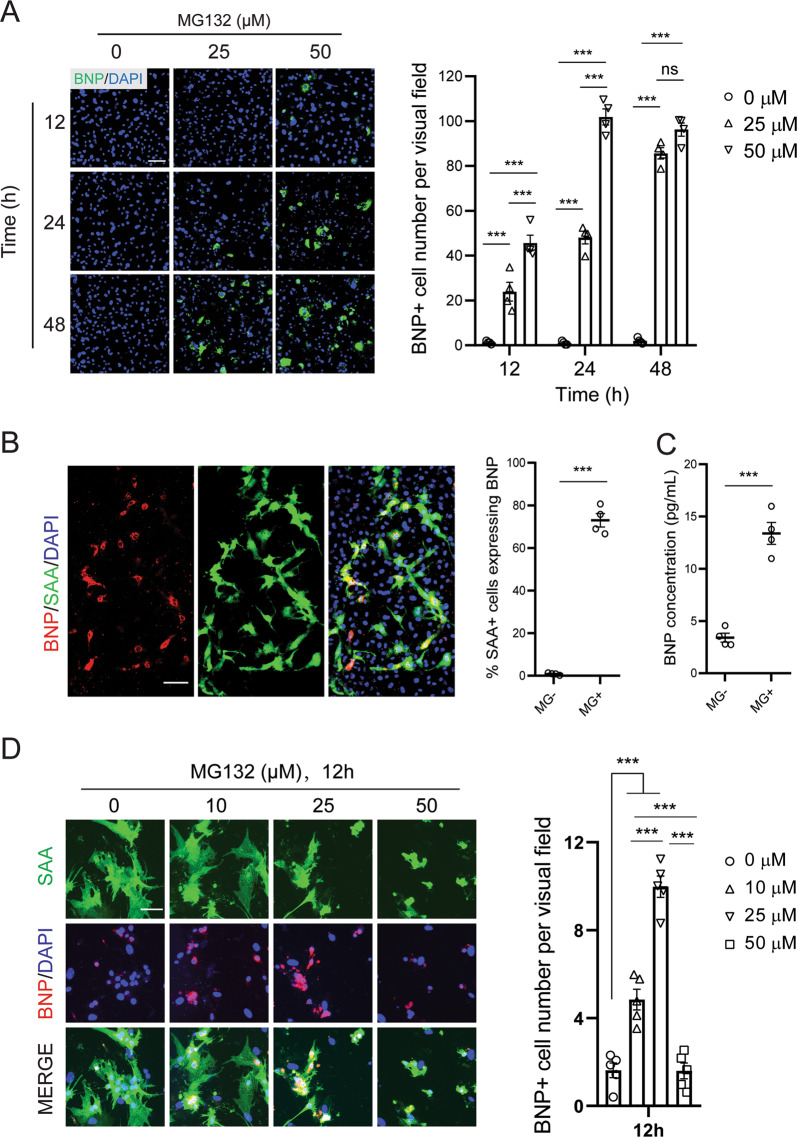
Proteasome inhibition induces brain natriuretic peptide (BNP) expression in cardiomyocytes. **a** Representative images and quantification of cells expressing BNP in response to two concentrations of MG132 at three time points in heterogeneous cell culture. Cell numbers were counted in 10 random areas of each culture coverslip using an eyepiece grid at a magnification ×40. *n* = 4 independent culture. **b** Representative images and quantification of sarcomeric α-actinin (SAA) + cardiomyocytes (green) expressing BNP (red) after 24-h treatment of 25 μM MG132 in heterogeneous cell culture. Note that all BNP + cells are SAA+. *n* = 4 independent culture. **c** Quantification of extracellular BNP concentrations measured from cell culture supernatants after cells were treated with or without 25 μM MG132 for 24 h. *n* = 4 independent culture. **d** Representative images and quantification of SAA+ cells expressing BNP in response to 12-h treatment of MG132 in homogeneous cardiomyocyte culture. Cell numbers were counted in 10 random areas of each culture coverslip using an eyepiece grid at a magnification ×100. *n* = 5 independent culture. Data represent mean ± S.E.M. ***P < 0.001, ns not significant; one-way ANOVA with Tukey’s post hoc analysis in (**a**, **d**), Student’s *t*-test in (**b**, **c**). Scale bar represents 50 μm.

Next, we sought to understand how this survival effect of cardiomyocytes was initiated. Two hypotheses were proposed. One assumed that the anti-apoptosis response is directly triggered by MG132 in noncardiomyocytes, while the other assumed that it was mediated by MG132-stimulated cardiomyocytes. To explore whether the anti-apoptosis support was dependent on cardiomyocytes, we developed a cell passaging method to selectively eliminate all the cardiomyocytes from the heterogeneous cell culture, while the noncardiomyocytes were not affected. The conditioned media were collected from these cultures after MG132 treatment and their activities on homogeneous cardiomyocytes were tested (Supplementary Fig. 2A). However, the conditioned media did not rescue the apoptotic cardiomyocytes (Supplementary Fig. 2B), suggesting that the stimulation of MG132 on only noncardiomyocytes was not able to provide anti-apoptosis support to cardiomyocytes. Hence, we hypothesized that the anti-apoptosis support from noncardiomyocytes was triggered by specific extracellular signaling molecule secreted by MG132-stimulated cardiomyocytes. Natriuretic peptides including ANP and BNP are secreted hormones best known for their cardiomyocyte-specific induction in response to various pathological insults, and NPR-A, the receptor for ANP and BNP, was ubiquitously present on cell surfaces of all the cardiac cell types^20^. To investigate whether either natriuretic peptide served as the extracellular signal for the communication between cardiomyocytes and noncardiomyocytes, immunostaining analyses against ANP and BNP were performed in heterogeneous cell culture exposed to MG132. Few cells expressed either ANP or BNP in the absence of MG132, whereas BNP expression was remarkably induced by MG132 in a time- and dose-dependent manner (Fig. 3a). In contrast, ANP expression was not induced by MG132 (Supplementary Fig. 3A). Co-labeling BNP with each cardiac cell type marker validated that BNP was exclusively expressed in SAA + cardiomyocytes, accounting for about 70% of total cardiomyocytes (Fig. 3b). In consistent with the intracellular BNP induction, enzyme linked immunosorbent assay targeting BNP confirmed an increased level of extracellular BNP in MG132-treated culture supernatant (Fig. 3c). These data demonstrated that cardiomyocytes specifically synthesized and emitted BNP in response to MG132. Interestingly, despite of the concurrent apoptosis, BNP was also significantly induced by MG132 in homogeneous cardiomyocyte culture (Fig. 3d), indicating that instead of being derived from a secondary feedback from noncardiomyocytes, the BNP expression in cardiomyocytes was directly activated by MG132.

MG132 is a peptide aldehyde targeting not only proteasome but also cathepsins and calpain^35^. To exclude the possibility that the BNP inducing effect was derived from inhibition of cathepsins and calpain, cells were treated with other two types of proteasome inhibitors as well as three types of cathepsins and calpain inhibitors. Proteasome inhibitors Bortezomib and Delanzomib resulted in similar BNP induction in cardiomyocytes as MG132. In contrast, none of the cathepsins and calpain inhibitors including Pepstatin A, Leupeptin hemisulfite or E-64-D induced BNP expression in cardiomyocytes (Supplementary Fig. 3B). These data strongly suggest that proteasome inhibition is indeed the cause of BNP induction in cardiomyocytes.

### Blockade of BNP receptor abolishes the survival effect of cardiomyocytes in heterogeneous cell culture

**Fig. 4:**
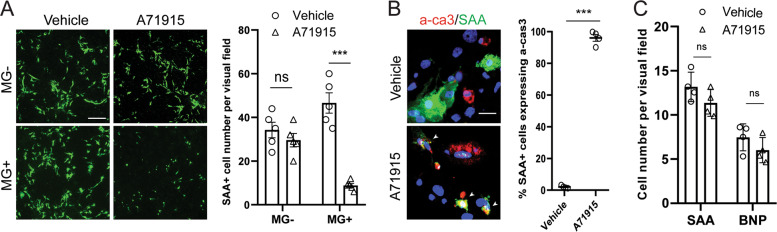
Extracellular brain natriuretic peptide (BNP) mediates the survival of cardiomyocytes in response to proteasome inhibition. **a** Representative images and quantification of sarcomeric α-actinin (SAA)+ cardiomyocytes (green) with or without treatments of BNP receptor inhibitor A71915 and MG132 (MG) in heterogeneous cell culture. *n* = 5 independent culture. Scale bar represents 50 μm. **b** Representative images and quantification of SAA + cardiomyocytes (green) co-labeling with active caspase 3 (a-ca3; red) treated with MG132 or MG132 and A71915. Cardiomyocytes undergoing apoptosis were indicated by white arrows. Cell nuclei were marked with 4′,6-diamidino-2-phenylindole (blue). *n* = 4 independent culture. Scale bar represents 20 μm. **c** Quantification of SAA+ cell number and BNP + cell number in homogeneous cardiomyocyte culture treated with MG132 or MG132 and A71915. *n* = 5 independent experiments. Scale bar represents 50 μm. Cell numbers were counted in 10 random areas of each culture coverslip using an eyepiece grid at a magnification ×40 in **a** and at ×100 in **c**. Data represent mean ± S.E.M. ***P < 0.001, ns not significant; Student’s *t*-test.

We then asked whether the extracellular BNP released by cardiomyocytes was associated with the survival of cardiomyocytes in heterogeneous cell culture. To block the reception of extracellular BNP, cells were pre-treated with a competitive NPR-A antagonist A71915^36^ before MG132. The number of SAA + cardiomyocytes was not affected by the treatment of A71915 in the absence of MG132, but was greatly decreased in the presence of MG132 (Fig. 4a). Importantly, nearly all the SAA + cardiomyocytes showed shrinkage morphology and expressed a-cas3 after exposed to A71915 and MG132 (Fig. 4b). These results clearly demonstrated that the survival of cardiomyocytes in heterogeneous cell culture was associated with the receiving of BNP in extracellular environment. Conversely, neither the survival of cardiomyocytes nor the BNP induction was affected by blockade of NPR-A in homogeneous cardiomyocyte culture under proteasome inhibition (Fig. 4c), suggesting that the autocrine receiving of BNP signal by cardiomyocytes did not contribute to the survival effect. Taken together, while the exact receiving cell population and downstream effectors are as yet unknown, it is plausible to suggest that a paracrine signaling mediated by BNP between cardiomyocytes and noncardiomyocytes is essential for cardiomyocytes to survive proteasome inhibition.

### Surviving cardiomyocytes are not derived from cardiac progenitor cell (CPC) proliferation or differentiation

**Fig. 5:**
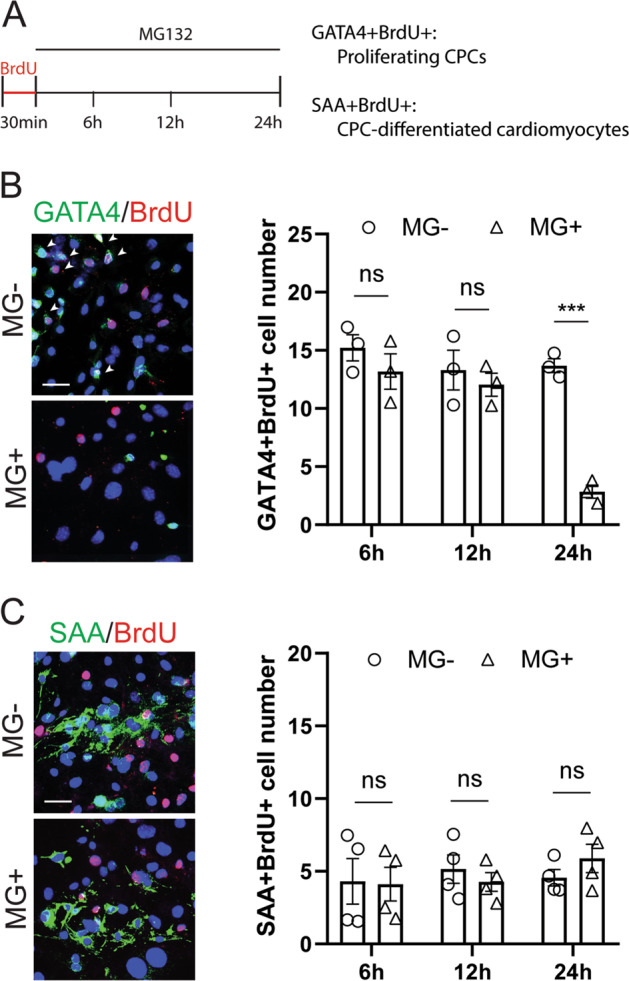
No contribution of cardiac progenitor cell (CPC) proliferation and/or differentiation to cardiomyocytes survived from proteasome inhibition. **a** Schematic of CPC fate-tracing procedure. 5-bromo-2′-deoxyuridine (BrdU) was introduced into cardiac cells for 30 mins to label all the proliferating cells. Cells were fixed at three time points (6, 12, and 24 h) within 25 μM MG132 (MG) treatment. **b** Representative images and quantification of proliferating CPCs which were double positive for BrdU and CPC marker GATA binding protein 4 (GATA4) with or without MG132 treatment. Images represent cells fixed 24 h after MG132. Arrow heads indicate double-positive cells. **c** Representative images and quantification of CPC-derived cardiomyocytes which were double positive for BrdU and sarcomeric α-actinin (SAA) with or without MG132 treatment. Images represent cells fixed 24 h after MG132. Arrow heads indicate double-positive cells. Cell numbers were counted in 10 random areas of each culture coverslip using an eyepiece grid at a magnification ×100. *n* = 3 independent experiments. Data represent mean ± S.E.M. ***P < 0.001, ns not significant; Student’s t-test. Scale bar represents 20 μm.

Besides four main cardiac cell populations, the heterogeneous cell culture also contained ~20% unmarked cells, which were likely to be undifferentiated or immature CPCs. Bielmann and colleagues have reported that CPCs in neonatal mouse hearts were able to proliferate and differentiate into new cardiomyocytes in response to exogenous BNP^26^. As BNP expression was induced by proteasome inhibition, we asked whether the surviving cardiomyocytes were at least partially derived from CPC proliferation and/or proliferation. To elucidate this possibility, we performed a BrdU-based fate tracing analysis of proliferating CPCs (Fig. 5a). All proliferating cells were labeled by short-term incubation with BrdU before MG132 treatment. During MG132 treatment, cells were fixed at three-time points to monitor the fate dynamics of BrdU-labeled cells. CPCs that did not undergo differentiation were identified as BrdU-labeled cells positive for CPC marker GATA4, whereas CPC-derived cardiomyocytes were identified as BrdU-labeled cells positive for SAA. Within 24-h MG132 treatment, the number of cells expressing both GATA4 and BrdU gradually decreased (Fig. 5b), suggesting that undifferentiated CPC population were not expanded but rather reduced, likely due to CPC apoptosis. In contrast, nearly no SAA + cardiomyocytes were found positive for BrdU during MG132 treatment (Fig. 5c), ruling out the hypothetical CPC differentiation toward new cardiomyocytes. Hence, we concluded that CPC proliferation or differentiation did not contribute to the maintenance of cardiomyocytes in response to proteasome inhibition.

### REST repressor complex mediates *Nppb* gene expression

**Fig. 6:**
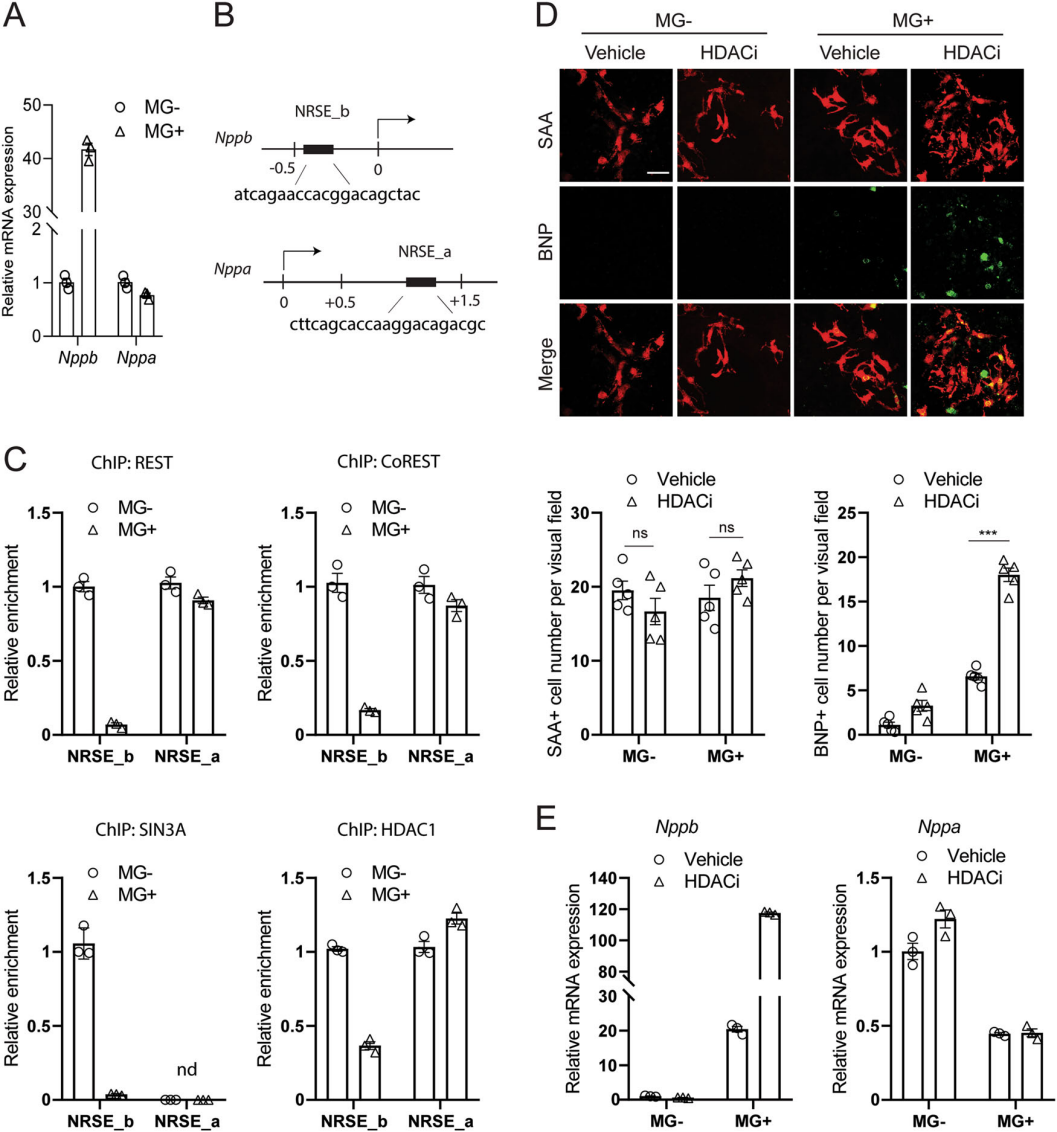
Association of *Nppb* gene expression with REST repressor complex. **a** Transcription of *Nppb*, but not *Nppa* was up-regulated upon MG132 (MG), evidenced by real-time quantitative PCR analysis. **b** Schematic of positions and consensus sequences of REST binding site (NRSE) on mouse *Nppb* and *Nppa* gene promoter and 3’ untranslated region, respectively. Position 0 represents transcription starting site. Unit of DNA is kilo base pair. **c** Chromatin immunoprecipitation (ChIP) analyses to determine recruitments of REST and its repressor complex components CoREST, SIN3A, HDAC1 on *Nppb* and *Nppa*. Cells were treated with or without 25 μM MG132 for 12 h. Immunoprecipitated DNA fragments were subjected to real-time quantitative PCR analyses using primers flanking NRSE on *Nppb* (NRSE_b) and on *Nppa* (NRSE_a). **d** Representative images and quantification of cells expressing sarcomeric α-actinin (SAA) and brain natriuretic peptide (BNP) after heterogeneous cell culture were treated with MG132 and a cocktail of two HDAC inhibitors (HDACi) Trichostatin A and valproic acid for 12 h. The HDACi was treated 1 h before MG132. Note that BNP intensity were clearly increased and more SAA + cardiomyocytes started to express BNP due to HDAC inhibition. Scale bar represents 20 μm. Cell numbers were counted in 10 random areas of each culture coverslip using an eyepiece grid at a magnification ×100. *n* = 5 independent experiments. Data represent mean ± S.E.M. ***P < 0.001, ns not significant; Student’s *t*-test. **e** Real-time quantitative PCR analysis showing mRNA expression of *Nppb*, but not *Nppa*, was further up-regulated by HDACi. For all PCR experiments, three independent experiments with very similar results were performed and one representative chart was presented.

Given that the induction of BNP in cardiomyocytes is the key event to initiate the anti-apoptosis support from noncardiomyocytes, we sought to investigate the molecular mechanism underlying this process. Consistent with the change of protein level, real-time PCR analysis showed that the *Nppb* expression was greatly up-regulated by MG132, whereas *Nppa* expression was not (Fig. 6a). Previous studies have identified REST as a key negative regulator for the transcription of both genes^34^. To explore whether REST was also involved in MG132-induced *Nppb* expression, we predicted one REST binding site (NRSE_b) on mouse *Nppb* promoter from position −413 to −393, and one (NRSE_a) on mouse *Nppa* 3’ untranslated region from position +1331 to +1352 based on the consensus sequence of neuron-restrictive silencer element (Fig. 6b). Both regions have been confirmed as bona fide repressive domains for *Nppb* and *Nppa* expression in previous studies^32,37^. ChIP assays were performed to examine the occupancy dynamics of REST and its co-repressors CoREST, SIN3A and HDAC1 on these two regions in response to MG132. We found that the bindings of all four molecules were significantly decreased at NRSE_b in MG132-treated cells compared to non-treated control, indicating that MG132 induced the disassociation of REST repressor complex from the REST binding site on *Nppb* promoter. In contrast, MG132 did not alter the bindings of REST, CoREST and HDAC1 at NRSE_a, and that of SIN3A was below the detection threshold (Fig. 6c). These data revealed that proteasome inhibition differentially regulated the enrichments of REST repressor complex on *Nppb* and *Nppa*. To further establish a link between REST repressor complex and gene expression, we tested whether the transcription of *Nppb* and *Nppa* would be affected by the manipulation of REST repressor complex component HDAC1. Trichostatin A and valproic acid are pharmaceutical inhibitors of HDAC1. We pre-treated cells with a cocktail of both inhibitors (HDACi) before MG132 to induce an incomplete function of REST repressive complex. As expected, the number of SAA + cells was not further increased, but more cells started to express BNP with stronger BNP immunoreactive intensity (Fig. 6d). *Nppb* expression was profoundly up-regulated in HDACi and MG132-treated cells than in MG132-treated cells, whereas *Nppa* expression was not altered (Fig. 6e). These data demonstrated a direct association of *Nppb* transcription with the activity of REST repressor complex on *Nppb* promoter. Taken together, these results provide mechanistic evidence that REST and its repressor complex components are important regulators of proteasome inhibition-activated BNP expression and survival of cardiomyocytes, thereby serving as potential targets of manipulation by epigenetic drugs.

## Discussion

Cardiac UPS dysfunction is originated from extrinsic stimuli of pathological conditions as well as intrinsic cues of aging and other physiological stresses. Proteasome inhibition and decline of proteasome activity have been shown to be associated with cardiomyopathies^13,38,39^, suggesting that cardiomyocytes are particularly prone to proteotoxicity. Indeed, we showed that cardiomyocytes cultured in homogeneous population were as vulnerable as, if not any more than, other noncardiomyocyte cell types such as vascular smooth muscle cells and endothelial cells to MG132-induced apoptosis. This result is consistent with several studies showing severe apoptosis effect of MG132 on purified culture of rodent cardiomyocytes^10,40^. However, unexpected survival effect of cardiomyocytes but not other three noncardiomyocyte populations was observed in the heterogeneous cell culture. Based on these findings, it is reasonable to deduce that the survival effect of cardiomyocytes comes from the anti-apoptosis support of neighboring noncardiomyocytes. The conclusion is further augmented by experiments showing that the survival effect could be diminished by blockade of extracellular BNP reception. This previously unraveled survival pathway of cardiomyocytes raises several intriguing questions. First, whether all the noncardiomyocyte populations contributes to the survival effect or it comes from only one specific population. Future study co-culturing cardiomyocytes with each individual noncardiomyocyte population will clarify this. Second, what kinds of anti-apoptosis support are responsible for the survival effect. Candidates include paracrine signalings mediated by cytokines and growth factors from cardiac fibroblasts^41^, angiocrines from endothelial cells^42^, or direct cell-cell interactions. Third, whether this survival effect is specific to proteasome inhibition or it is common in other cardiac stresses that are able to induce BNP expression in cardiomyocytes. In fact, BNP is inducible by various kinds of stimuli^10,33,40,43,44^. Re-visiting those experiments with the culture methods used in this study would provide new insights into the pathophysiological basis of those stresses.

Our finding also highlights the importance of noncardiomyocytes for the prevention of cardiomyocyte apoptosis. Given their vast majority of composition in the heart, the cardioprotective functions of noncardiomyocytes are likely to be underestimated^45^. Taking noncardiomyocytes into consideration might resolve differences regarding opposite outcomes from similar experimental or therapeutical purposes. For instance, proteasome inhibition is generally considered harmful to cardiac functions. However, evidence also exists implicating proteasome inhibition in prevention and reversal of maladaptive hypertrophy and cardiac remodeling^40,46,47^. Several studies have suggested that the controversy could be due to the extent of proteasome inhibition or its effects on noncardiomyocyte compartment^5,48^. Our work supports the notion that the status of local noncardiomyocytes and their responsiveness to BNP might cause great differences to the end outcome of cardiomyocytes. For instance, in pathological conditions which had little damage to noncardiomyocytes, the cardiomyocytes might behave much better than in those conditions with prevalent noncardiomyocyte damage. It would be interesting to measure and compare the BNP expression and the status of noncardiomyocytes in various pathological conditions to elucidate this hypothesis.

BNP is mainly regarded as a cardioprotective hormone in the diagnosis and treatment of heart diseases. We demonstrate for the first time a paracrine role of BNP for the survival of cardiomyocytes. This conclusion is step-by-step deduced by conducting several following experiments. First, the conditioned medium experiment confirms that MG132 did not directly stimulate noncardiomyocytes to release essential factors for the survival effect of cardiomyocytes. Combined with the fact that the survival effect was associated with extracellular release of BNP in cell supernatants, we come to conclusion that it is BNP that motivates the noncardiomyocytes to provide anti-apoptosis support to cardiomyocytes. Second, fate-tracing experiment confirms that no new cardiomyocytes were generated by MG132, thus the number of cardiomyocytes is not maintained by complement of new cardiomyocytes derived from CPCs. Although an increase of extracellular BNP was detected in the culture supernatants, it was much lower than the exogenous BNP concentration (1 μM) Bielmann and colleagues treated to neonatal cardiomyocytes^26^, which might explain the discrepancy of our results and theirs. Third, the autocrine role of BNP is excluded by experiments that blocked BNP receptor in homogeneous cardiomyocyte culture. Unlike the survival effect, the induction of BNP in cardiomyocytes was independent of noncardiomyocytes. In both heterogeneous and homogeneous cardiomyocyte culture, MG132 induced *Nppb* transcription and BNP protein expression. However, the increased BNP failed to rescue any cardiomyocytes in homogeneous cardiomyocyte culture, suggesting that BNP does not directly affect on cardiomyocytes.

We further provide mechanistic insight into the selective up-regulation of BNP. The paralogous feature of *Nppa*-*Nppb* cluster offers wide choices for regulatory elements, transcription factors and epigenetic regulators to orchestrate its regulation. The transcriptional activation of *Nppb* and *Nppa* has been shown to share common regulators such as GATA4 and GATA6^49^. Different transcriptional regulatory mechanisms underlying the induction of *Nppb* and *Nppa* has also been reported^29^. REST repressor complex is preferentially assessed, as REST has a widely studied database in cardiac gene expression^33,34,50–53^, a definite DNA consensus sequence to predict^54^, and versatile functions of co-repressor components^55,56^. We provide evidence that the transcriptional re-activation of *Nppb* coincided with a de-recruitment of REST and several repressor complex components from the consensus sequence of REST binding site on *Nppb* promoter. The fact that inhibition of HDAC activity by HDACi further activated *Nppb* transcription further validated the results from ChIP experiments. Our finding is consistent with previous study showing the de-recruitment of REST and HDAC1 from *Nppb* promoter in endothelin-1 stimulated cardiomyocytes^32^, suggesting that REST repressor complex might be a common regulator for stress-stimulated BNP expression.

**Fig. 7:**
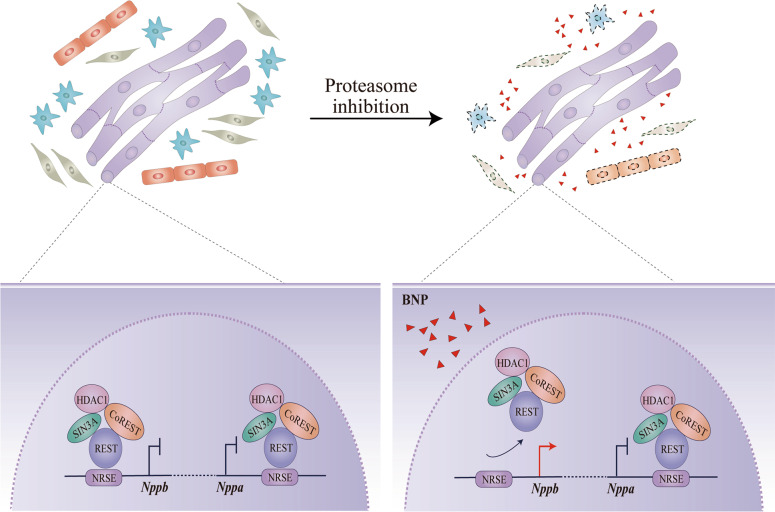
Schematic summary of the paracrine function of BNP. In a co-culture of four major cardiac cell populations, proteasome inhibition induces apoptosis in cardiac fibroblasts (grey), endothelial cells (red), vascular smooth muscle cells (blue), but not cardiomyocytes (purple). Cardiomyocytes synthesize and secret brain natriuretic peptide (BNP; red triangles) in response to proteasome inhibition. BNP is received by neighboring noncardiomyocytes and motivates them to provide anti-apoptosis support to cardiomyocytes. On molecular level, repressor element-1 silencing transcription factor (REST) repressor complex differentially regulates the paralogous *Nppb*-*Nppa* cluster in response to proteasome inhibition. The transcriptional activation of BNP-encoding gene *Nppb* is dependent on de-recruitment of REST repressor complex from REST binding site (NRSE) on *Nppb* promoter. In contrast, the atrial natriuretic peptide encoding gene *Nppa* is maintained in silencing status without de-recruitment of the complex from *Nppa* NRSE.

In summary, the current study demonstrates a novel crosstalk between cardiomyocytes and noncardiomyocytes, which efficiently prevents cardiomyocyte apoptosis from proteasome inhibition. The crosstalk is initiated by cardiomyocyte-released BNP paracrine signaling, which is under regulation of REST repressor complex (Fig. 7).

## Supplementary information

Suppl.figure legends_clean version

Suppl. Figure 1

Suppl. Figure 2

Suppl. Figure 3
