# Supplementary material for: Crosstalk between cardiomyocytes and noncardiomyocytes is essential to prevent cardiomyocyte apoptosis induced by proteasome inhibition
Source: Cell Death Dis. 2020 Sep 19;11(9):783. doi: 10.1038/s41419-020-03005-8 (PMC7502079; doi:10.1038/s41419-020-03005-8)
Supplement: Supplementary file 1 — Suppl.figure legends_clean version [file 41419_2020_3005_MOESM1_ESM.docx]

**Supplementary Fig. 1** (A) MG132 efficiency on induction of polyubiquitinated proteins was confirmed by western blotting using anti-ubiquitin antibody. (B) Cell composition of neonatal mouse heart culture. Left, representative images of cells immunostained for four cardiac cell type markers. Right, quantification of each cell type population. Scale bar represents 20 μm. n=5 independent culture. Data represent mean ± S.E.M.

**Supplementary Fig. 2** Assessment of the survival effect of conditioned media (CM) derived from MG132-stimulated noncardiomyocyte culture on homogeneous cardiomyocytes. Noncardiomyocyte culture was treated with 10 μM MG132 for 4 hours. Cells were washed once extensively with culture media to avoid any carryover of MG132. CM were made by incubation of cells with fresh culture media for another 4 hours and filtered through 0.45μm filter and stored in -80℃. Homogeneous cardiomyocytes were cultured in conditioned media and further stimulated by 10 μM MG132 for 12 hours. (A) Experimental schematic. (B) Quantification of sarcomeric α-actinin (SAA)+ cardiomyocytes. Cell numbers were counted in 10 random areas of each culture coverslip using an eyepiece grid at a magnification 100X. n=4 independent culture. Data represent mean ± S.E.M. *** *P* < 0.001, ns, not significant; one-way ANOVA with Tukey's post hoc analysis.

**Supplementary Fig. 3** (A) Atrial natriuretic peptide (ANP) expression is not induced by proteasome inhibition. Representative images and quantification of ANP-expressing cells treated with or without MG132 for various time periods were shown. Scale bar represents 50 μm. n=5 independent culture. Data represent mean ± S.E.M. (B) Proteasome inhibitors but not cathepsins and calpain inhibitors induced brain natriuretic peptide (BNP) expression in sarcomeric α-actinin (SAA)-labeled cardiomyocytes. Heterogeneous cell culture were treated with three types of proteasome inhibitors (MG132, Bortezomib and Delanzomib) and three types of cathepsins and calpain inhibitors (Pepstatin A, Leupeptin hemisulfite and E-64-D) for 24 hours. Scale bar represents 50 μm.
