# Supplementary figures and images for: Crosstalk between cardiomyocytes and noncardiomyocytes is essential to prevent cardiomyocyte apoptosis induced by proteasome inhibition
Source: Cell Death Dis. 2020 Sep 19;11(9):783. doi: 10.1038/s41419-020-03005-8 (PMC7502079; doi:10.1038/s41419-020-03005-8)

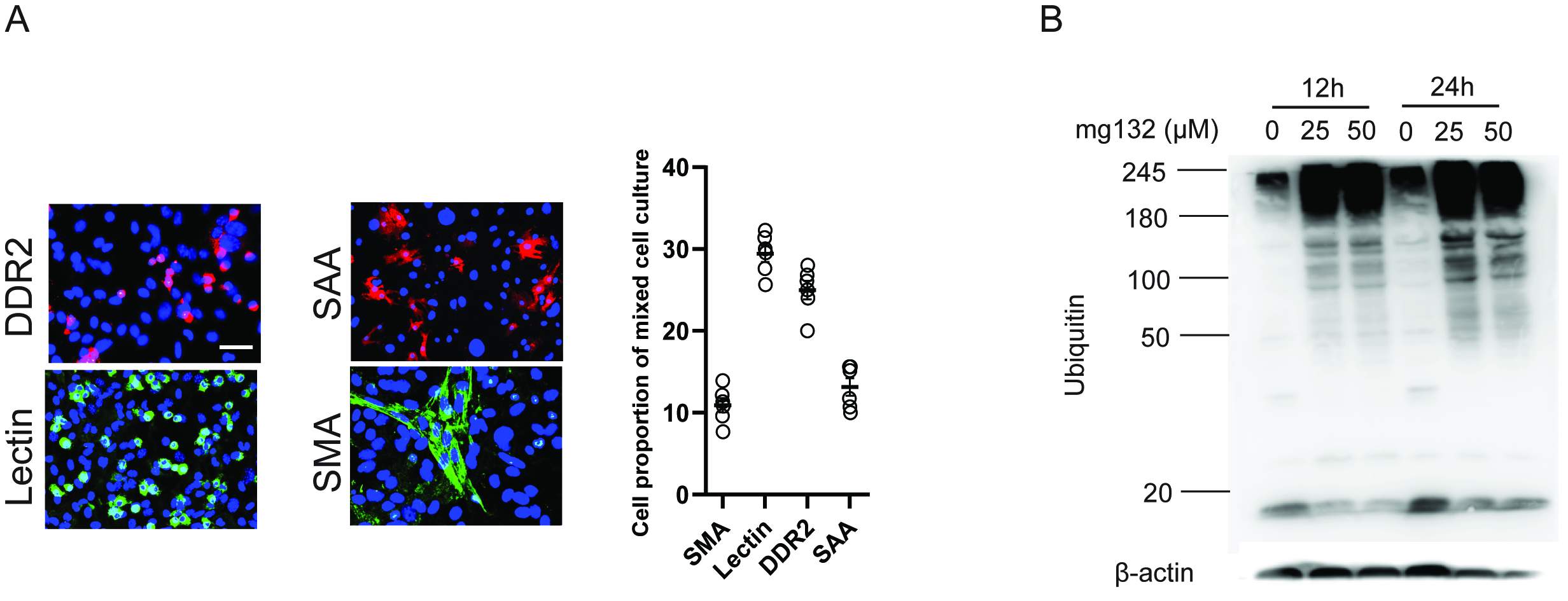

Supplement: Supplementary file 2 — Suppl. Figure 1 [file 41419_2020_3005_MOESM2_ESM.tif]

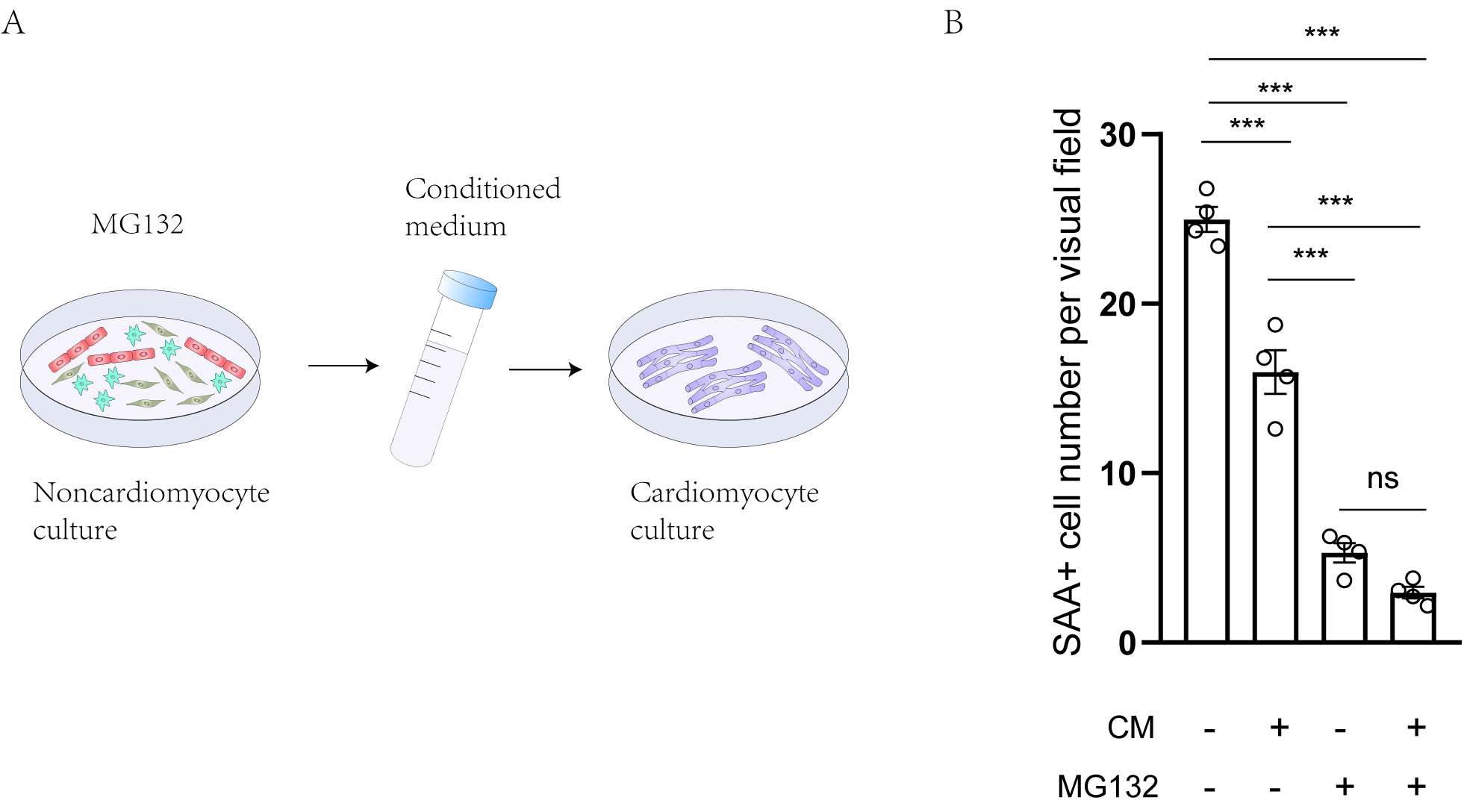

Supplement: Supplementary file 3 — Suppl. Figure 2 [file 41419_2020_3005_MOESM3_ESM.tif]

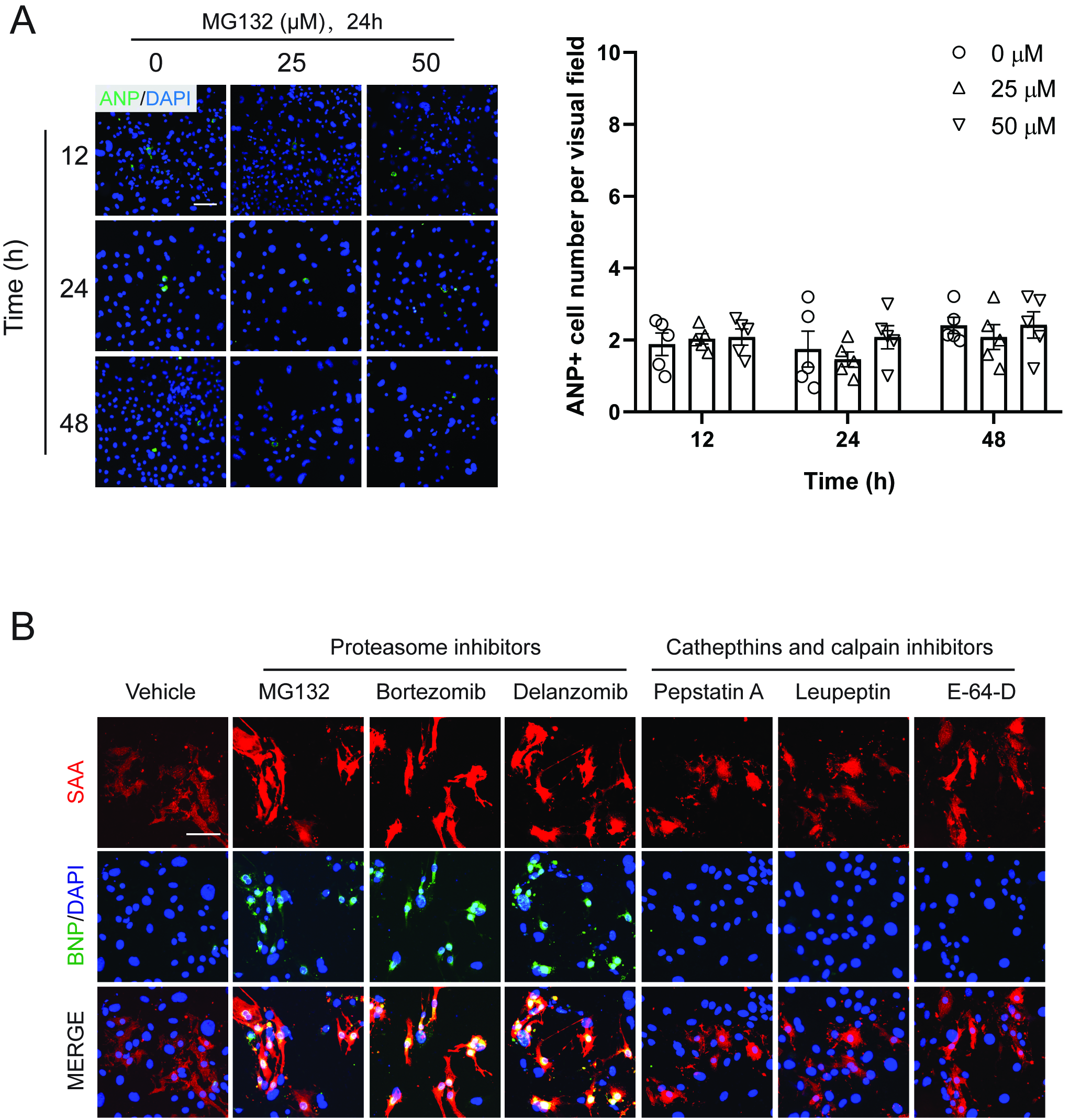

Supplement: Supplementary file 4 — Suppl. Figure 3 [file 41419_2020_3005_MOESM4_ESM.tif]
